# Surface Modification of Carbon Nanotubes with an Enhanced Antifungal Activity for the Control of Plant Fungal Pathogen

**DOI:** 10.3390/ma10121375

**Published:** 2017-11-30

**Authors:** Xiuping Wang, Zilin Zhou, Fangfang Chen

**Affiliations:** 1 College of Life Science and Technology, Hebei Normal University of Science and Technology, Qinhuangdao 066000, China; wangxiuping0721@163.com; 2 CAS Key Laboratory of Plant Germplasm Enhancement and Specialty Agriculture, Wuhan Botanical Garden, Chinese Academy of Sciences, Wuhan 430074, China; zhouzilin16@mails.ucas.ac.cn

**Keywords:** MWCNTs, surface modification, antifungal activities, plant protection

## Abstract

The addition of surface functional groups to multi-walled carbon nanotubes (MWCNTs) expands their application in engineering, materials, and life science. In the study, we explored the antifungal activities of MWCNTs with different surface groups against an important plant pathogenic fungi *Fusarium graminearum*. All of the OH-, COOH-, and NH_2_-modified MWCNTs showed enhanced inhibition in spore elongation and germination than the pristine MWCNTs. The length of spores decreased by almost a half from 54.5 μm to 28.3, 27.4, and 29.5 μm, after being treated with 500 μg·mL^−1^ MWCNTs-COOH, MWCNTs-OH, and MWCNTs-NH_2_ separately. Furthermore, the spore germination was remarkably inhibited by surface-modified MWCNTs, and the germination rate was only about 18.2%, three times lower than pristine MWCNTs. The possible antifungal mechanism of MWCNTs is also discussed. Given the superior antifungal activity of surface modified MWCNTs and the fact that MWCNTs can be mass-produced with facile surface modification at low cost, it is expected that this carbon nanomaterial may find important applications in plant protection.

## 1. Introduction

Carbon nanotubes (CNTs) are considered one of the most popular types of nanomaterials with unique morphologies and surface properties and have been intensively studied for various applications in bionanotechnology, including drug and gene delivery, tissue engineering, plant-technology [1,2], and other biomedical applications [3,4,5,6,7,8]. In recent years, CNTs have been found to have an active antibacterial activity and garnered a significant research interest around the use of nanotechnology-based approaches for agricultural system and plant protection [9,10,11,12]. A nanotube filter covered with a thin layer of single-walled carbon nanotubes (SWCNTs) are demonstrated to be effective in removing viral and bacterial pathogens [13,14,15]. Pristine SWCNTs dispersed in a biocompatible surfactant solution exhibited strong bactericidal activity against both gram-positive and gram-negative bacteria [16]. Lately, an exceptional application of CNTs in controlling plant pathogens in biological science has been described [17]. From the toxicological point of view, single-walled carbon nanotubes have higher antimicrobial properties than multi-wall carbon nanotubes (MWCNTs) [18]. At the same time, our previous studies verified that CNTs displayed superior inactivation effects on the copper-resistant plant pathogenic microorganisms *Ralstonia solanacearum*, *Fusarium graminearum*, and *F. oxysporum* [19,20]. These findings implied that CNTs may be applied to phytopathogen control in plant protection because of their superior antimicrobial activity.

However, a crucial step toward the application of CNTs in the plant science is to regulate their impacts on biological systems. The use of CNTs is currently considered with apprehension owing to their yet undefined safety profile and their potential environmental and health risks, especially given their structural resemblance to asbestos fibers [21]. It has been proven that surface functional groups on multiwalled CNTs (MWCNTs) reduce their immune perturbations in mice and in macrophages and improve the colloidal properties of the CNT dispersions without changing their inherent antibiosis performance [22,23]. For example, well-functionalized CNTs are safe to animal cells, while CNTs without functionalization show severe toxicity to animal or human cells even at low doses [24]. Moreover, several studies have shown that the surface functional groups of CNTs are the critical factors that affect the overall antibacterial effects of CNTs [25,26]. In contrast to the focused studies on antibacterial properties of CNTs, researches related to antibiosis activities of CNTs in the treatment of plant fungal infections are underemphasized. However, more than 80% of plant diseases are caused by pathogenic fungi. There are relatively few reports about the influence of surface chemistry and length of CNTs on fungi growth and propagation. Therefore, it is critical to study the antifungal properties of CNTs in plants to explore their potential antifungal activity.

In many cases, placing functionalized covalent and non-covalent chemical groups onto the surface of nanotubes can improve their biological performance [27]. For example, sugar with a terminal amino group used to modify SWCNTs can control their aqueous solubility and biological activity in binding assays with pathogens [28]. In additions, studies showed that SWCNTs with surface functional groups of –OH and –COOH displayed very strong bactericidal activity against both gram-positive and gram-negative bacteria, while similar functional groups of MWCNTs did not display antimicrobial action to either type of microbial cells [29]. Other studies have documented that functionalized SWCNTs (f-SWCNTs) have lower cytotoxicity to mammalian cells than SWCNTs [30,31]. Herein, we study the antifungal properties of CNTs against pathogenic fungal diseases with special emphasis on the effects of CNT surface chemistry modification and length on their antifungal activity. First, we studied the effects of different surface functional groups (–OH, –COOH and –NH_2_) of MWCNTs on the antifungal activity against *F. graminearum*, which causes head blight or scab in wheat. Second, we also discussed the potential antifungal mechanism of surface modified MWCNTs. MWCNTs were selected in this study, because they can be produced in mass-scale and low-cost. To the best of our knowledge, this is the first report to address this question. The results of this study will advance the application of MWCNTs as antimicrobial agents in plant protection.

## 2. Results

### 2.1. Surface Modification Effects of MWCNTs on Spore Length

**Figure 1:**
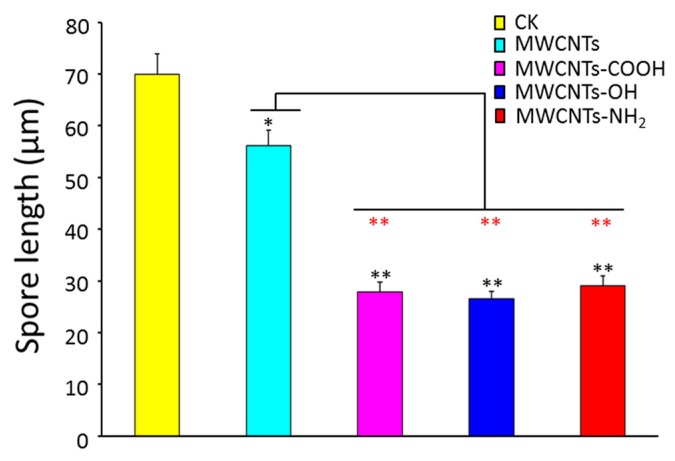
Effects of multi-walled carbon nanotubes (MWCNTs) modified with different groups (500 μg·mL^−1^) on the spore length. Error bars represent the standard deviation (*N* = 4). Black asterisk indicated significant differences within group consisting of Control (CK), MWCNTs, MWCNTs-COOH, MWCNTs-OH, and MWCNTs-NH_2_ based on analysis of variance using the GLM procedure with SAS system. GLM, General Linear Model; SAS, Statistics Analysis System. Red asterisk indicated significant difference between MWCNTs, MWCNTs-COOH, MWCNTs-OH, and MWCNTs-NH_2_, determined as described above. * *p* < 0.05; ** *p* < 0.01.

Fungal spores are specialized reproductive structures and play an important role in the dissemination of diseases [32]. As shown in Figure 1, the average length of normal spores is about 68.5 μm, and was reduced to 54.5, 28.3, 27.4, and 29.5 μm after being treated with MWCNTs, MWCNTs-COOH, MWCNTs-OH, and MWCNTs-NH_2_, respectively. The spore length was affected by MWCNTs—it was one-fifth shorter than the control group—and was significantly affected after it was treated with functional MWCNTs modified by the –OH group—It was almost three-fifths shorter than the control group on average, and one-half shorter than the group treated with pristine MWCNTs. Previous studies demonstrated that water is a major factor required for spore germination in the resumption of cellular metabolism and growth, and after water uptake by spore, swelling can significantly increase the volume of spores [32]; accordingly, the length of spore will be increased. In our experiment, the length of spores did not increase after being treated with CNTs, indicating that spores did not absorb water normally, and thus they could not germinate normally.

### 2.2. Surface Modification Effects of MWCNTs on Spore Germination

**Figure 2:**
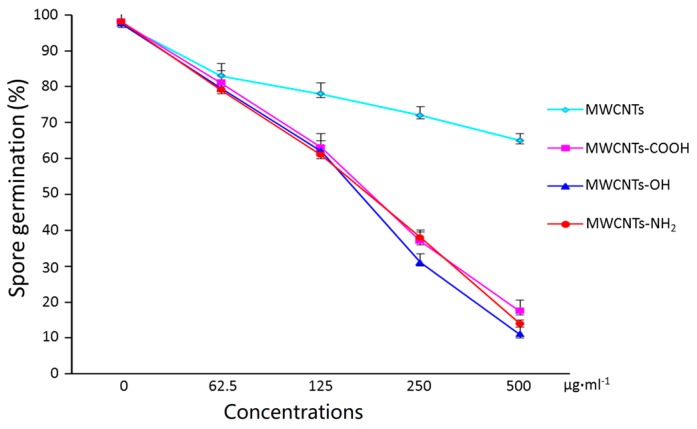
Effects of different functional groups of MWCNTs on the spore germination. Spores were germinated in distilled water at 28 °C in darkness at different concentrations of MWCNTs, MWCNTs-COOH, MWCNTs-OH and MWCNTs-NH_2_ dispersions. Germination was evaluated after incubation for 5 h. Error bars represent the standard deviation (*N* = 4).

Spore germination represents a pivotal step in the colonization of new environments by filamentous fungi [33]. As shown in Figure 2, we studied the effects of pristine MWCNTs and modified MWCNTs (surface modified by –OH, –COOH and –NH_2_) on *F. graminearum* spore germination. *F. graminearum* spores were incubated in dispersions of MWCNTs, MWCNTs-OH, MWCNTs-COOH, and MWCNTs-NH_2_ at the concentration from 62.5 to 500 μg·mL^−1^ for 5 h. MWCNTs caused a dose-dependent inhibitory effect on *F. graminearum* spore germination. When the spore germination rate reached 98.1% in the control, it was only 55.2% in the MWCNT group. Spore germination decreased by more than 30% at the highest dose of MWCNTs-COOH, -OH, and -NH_2_ tested (500 μg·mL^−1^). Figure S1 shows the corresponding photomicrograph of spore germination after treatment with MWCNTs, which visually confirmed that MWCNTs, especially modified MWCNTs, can effectively restrain the spore germination. This result suggests that the functional MWCNTs with –COOH, –OH, and –NH_2_ groups have stronger antifungal activity than pristine ones. From Figure 2, MWCNTs with –OH groups showed highest antifungal activity on spore germination among the three groups.

### 2.3. Surface Modification Effects of MWCNTs on Germination Pattern of Spores

**Figure 3:**
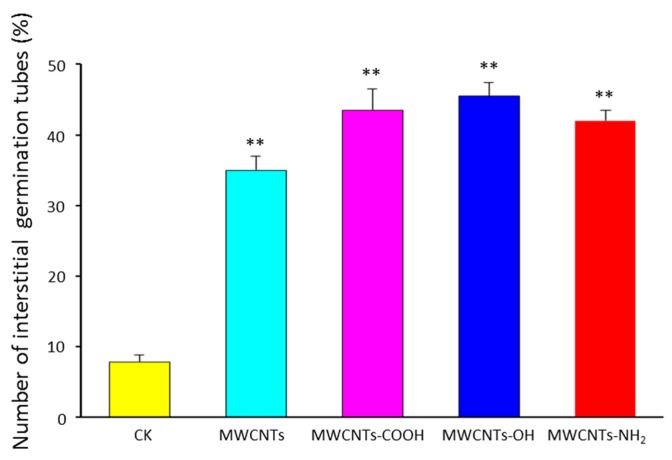
Effects of MWCNTs on the pattern of germ tube. The number of interstitial germ tubes was counted after 6 h of incubation at 28 °C in water (control) or water containing 500 μg·mL^−1^ MWCNTs. Error bars represent the standard deviation (*N* = 4). Where appropriate, statistical significance is indicated: ** *p* < 0.01.

*F. graminearum* typically has a bipolar germination pattern in which one germ tube emerges from each apical cell of the spore [32]. After treated with MWCNTs, there was a difference in the branching pattern of the germ tubes from conidia. The number of germ tubes originating from the terminal cells was significantly reduced (*p* < 0.05), especially when treated by surface functional MWCNTs with –COOH, –OH, and –NH_2_ groups at concentration of 500 μg·mL^−1^. As shown in Figure 3, 7.8% of the germ tubes emerged from interstitial cells in control group, but this number increased to 35.0%, 42.3%, 44.6%, and 38.5%, respectively, in MWCNTs-treated groups. Also, modified MWCNTs showed enhanced teratogenicity to *F. graminearum*. Figure S2 displays the representative microscopic images of spore germination after treatment with different surface functional MWCNTs, confirming that a larger proportion of interstitial germ tubes occurred when exposed to surface functional MWCNTs. As shown in Figure S2, long and normal germ tubes could be observed visually in the control samples. However, most germ tubes of the spores immersed in surface functional MWCNTs (500 μg·mL^−1^) developed from the side of spores.

### 2.4. Surface Modification Effects of MWCNTs on Persistence of Antifungal Activity

**Figure 4:**
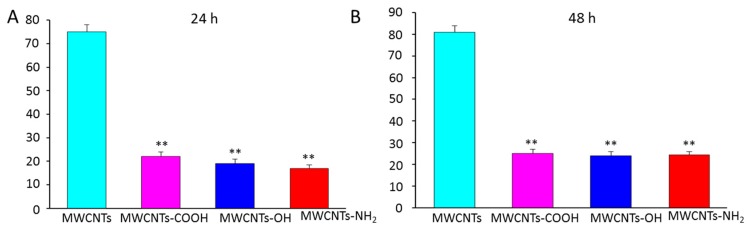
Effects of different functional MWCNTs on the germination rate at (**A**) 24 h and (**B**) 48 h, respectively. Error bars represent the standard deviation (*N* = 4). Where appropriate, statistical significance is indicated: ** *p* < 0.01.

We next examined the time-dependent antifungal behavior of pristine MWCNTs and functional MWCNTs. More effective and durable inhibitory activity toward spore germination was observed at 24 and 48 h in the groups treated with functional MWCNTs versus pristine nanotube. As shown in Figure 4, after treatment with functional MWCNTs, the germination rate was only about 25% even at 48 h. The groups treated with pristine MWCNTs had a germination rate of >70% at 24 h.

### 2.5. Length Effect of MWCNTs on Antifungal Activity

**Figure 5:**
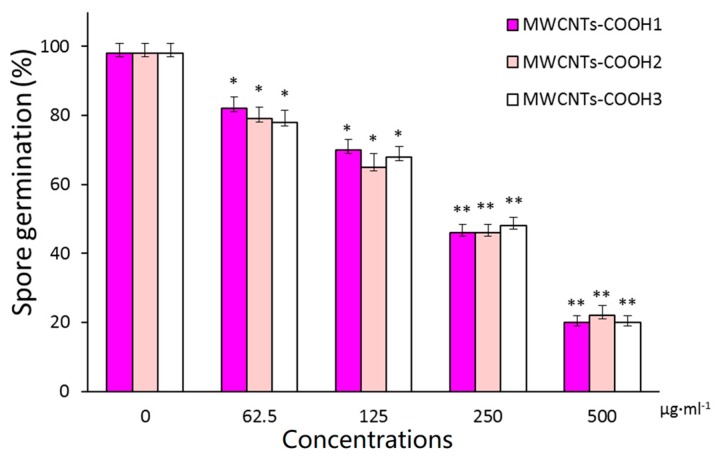
Antifungal effects of different length of MWCNTs-COOH on the germination rate of spores. Error bars represent the standard deviation (*N* = 4). Where appropriate, statistical significance is indicated: * *p* < 0.05; ** *p* < 0.01.

We also examined the influence of MWCNTs length on their antifungal activity. Three different lengths of MWCNTs-COOH were chosen as a model for this study. Figure 5 shows the germination rate of spores that were treated with different lengths of MWCNTs-COOH at different concentrations for 5 h. As shown in Figure 5, there is no significant difference in the germination rate of spores treated with MWCNTs-COOH_1_ (~50 μm in length), MWCNTs-COOH_2_ (10–30 μm in length), and MWCNTs-COOH_3_ (0.2–2 μm in length) at the same weight concentration.

A brief summary can be drawn for the antifungal activity of MWCNTs: (1) the surface chemistry of MWCNTs plays an important role in their antifungal activity; (2) the functional MWCNTs show stronger antifungal effects than pristine samples regardless of surface coating groups (–OH, –COOH, or –NH_2_); (3) the length of MWCNTs does not significantly affect their antifungal activity.

### 2.6. The Antifungal Mechanism of Surface Modification MWCNTs

**Figure 6:**
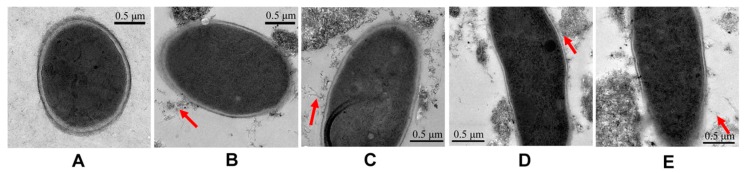

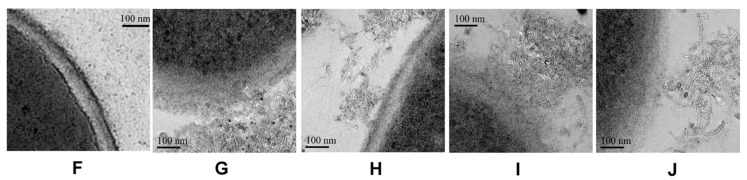
TEM images of spores after incubation with deionized (DI) water and MWCNTs. In the upper row are microscopic images of spores treated without (**A**) and with MWCNTs, MWCNTs-COOH, MWCNTs-OH, and MWCNTs-NH_2_ (**B**–**E**). The lower row shows partial magnification images of spores treated without (**F**) and with MWCNTs, MWCNTs-COOH, MWCNTs-OH, and MWCNTs-NH_2_ (**G**–**J**). The red arrows indicate MWCNTs around the spores and the magnified location of **B**–**E**.

To better understand the antifungal mode of MWCNTs, we examined how MWCNTs interacted with spores using TEM. In Figure 6B–E, a few clusters of pristine MWCNTs or functional MWCNTs accumulated around the spores. The tubular MWCNTs could be observed lying beside the spores (Figure 6G–J). These results indicate that the contact between spores and CNTs is a necessary condition for the inactivation of spores. Moreover, as shown in Figure 6, the spores in control group and MWCNTs-treated groups had basically complete cellular structure, indicating the integrity of the cell wall. It implicates that disrupting the cell wall with CNTs is not a major cause of spore inactivation.

## 3. Discussion

The volume of spore increases dramatically during swelling, which is essentially caused by water absorption [34]. This procedure is under the influence by temperature, culture solution, and protein nutrients. In this study, all the treatment experiments were carried out in distilled water at 28 °C, so the influences of temperature are excluded. Therefore, the enhanced inhibition of spore elongation is dominated by additional functional groups on the surface of MWCNTs which causes the significant decrease in spore size.

Noticeably, the MWCNTs have only mild inhibition on spore germination, but the modified MWCNTs show superior activity against *F. graminearum* spores. This is likely due to the marked difference in dispersity of the functional MWCNTs and pristine MWCNTs. Generally, the MWCNTs dispersions are unstable and containing larger aggregates which effectively block the interaction with spores. The dispersibility of MWCNTs can be improved by the functional groups on their surface [35]. When carboxyl, hydroxyl, amino, and epoxy groups are introduced onto the surface of the MWCNTs, they facilitate the formation of much more stable dispersions versus the hydrophobic pristine carbon planes [36]. Thus, the MWCNTs surface modified with –OH, –COOH, and –NH_2_ groups can form small and stable dispersions, which offers more opportunities to interact with spores than pristine MWCNTs.

Previous studies suggested that charge effect is a very important factor that affects the antibacterial efficiency of CNTs [24]. Here, the pH values of these incubation buffers are 7.0. At this pH, MWCNTs-OH are neutrally charged, while MWCNTs-COOH are negatively charged and MWCNTs-NH_2_ are positively charged. However, the antifungal activity of functional MWCNTs shows no significant difference between these three surface functional groups, indicating that the charge effect is not a crucial factor for antifungal efficiency of MWCNTs. It is distinct with findings obtained from bacterial cells, in which charge effects of MWCNTs change the interaction between bacterial cells and MWCNTs [24]. Normally, both the gram-positive and gram-negative cell walls have overall negative charges [24]. According to previous reports [15,37], the antibacterial mechanisms of CNTs are mainly due to destruction of cell wall integrity by direct contact with CNTs. Therefore, we suspect that the distinct performance of MWCNTs as antimicrobial against fungal spores or bacterial cells is attributed to the difference between their cell walls.

Meanwhile, we observed that a larger proportion of interstitial germ tubes occurred when exposed to MWCNTs at the dose of 500 μg·mL^−1^ MWCNTs. The results are consistent with the work of Harris et al. [32]. They have found that spores germinate preferentially from the lateral end under certain conditions, such as high glucose or other adverse environmental conditions. These results suggest that MWCNTs is an unfavorable factor for the growth and development of spores. It was shown that the pattern of germination requires the presence of functional microtubules, which may be responsible for the transport of key polarity factors for chitin deposition to specific sites. Thus, in our experiments, we speculate that the regulatory system for chitin deposition to specific sites may be disturbed after treated by MWCNTs [38]. Similar phenomena have been previously observed on other carbon nanomaterials. For instance, when *F. graminearum* spores were exposed to increasing concentrations of graphene oxide (GO), more interstitial germ tubes were noted and then the length of germ tube was inhibited, and GO treatment can remarkably reduce macroconidia viability ultimately [39].

A notable difference between the pristine MWCNTs and functional MWCNTs is the lack of dispersibility of pristine MWCNTs in most solvents owing to strong inter-tube van der Waals forces, and this has been an obstacle for their effective use in antifungal applications. However, this may be largely overcome by surface modification of the nanotube backbone, allowing the functional MWCNTs well dispersed in water to form a homogeneous solution. It is much more stable even after several days of storage [39]. These results suggest that the stability of the MWCNTs dispersions is critical to their antifungal activities.

One prior study on the length effects of SWCNTs on bacteria showed that the antimicrobial activity of SWCNTs increased with increasing length of SWCNTs [39]. In contrast to this observation, the present study (Figure 5) did not indicate any length effects of the MWCNTs on their antifungal activity, at least in the length range discussed here. One possible explanation is the inherent composition differences between fungi spores and bacteria. Fungal spores are usually dozens of microns long and have chitin cell walls, while bacteria are only a few microns long and have cell walls made of cellulose [40]. These size and morphology differences result in different interactions between spores or bacteria and CNTs. Due to smaller size of bacteria, CNTs are easily intertwined and packaged with them and damages their cell walls. The larger fungal spores are more resistant to damage because the CNTs only adhere to the spore rather than integrating with the cell wall. Furthermore, the main difference between the cell walls of fungi and bacteria is that the cell wall of fungi is mainly composed of chitin and cellulose, while the cell wall of bacteria is mainly composed of peptide polysaccharides. Fungal spores are more resistant to mechanical damage, because the main composition of their cell walls is chitin and cellulose, which are stronger than peptide polysaccharides. Thus, CNTs probably cannot damage the spore cell walls. This hypothesis was further verified by TEM imaging.

This finding is also in good agreement with our prior work in which the damage and disruption of the cell wall is not a dominating mechanism to interpret the antifungal activity of MWCNTs. The possible antifungal mechanism is the blockage of water channel imposed by surface-adsorbed MWCNTs [19]. During the exposure of spores to MWCNTs, the MWCNTs seemed to be more inclined to form aggregates with the largest involvement of spores. The MWCNTs aggregates absorbed on the spore surface and blocked the water channel. This caused a substantial loss of water involved in germination. The proposed mechanism could also be applied to interpret the effects of MWCNTs on spore length.

The plant disease Fusarium head blight (FHB) is caused by the fungal pathogen *F. graminearum* and is a serious plant disease world widely affecting wheat, corn, etc. [41]. Currently, the wheat industry has few effective materials for FHB control. In this work, we demonstrated that surface modified MWCNTs showed enhanced inhibition in spore germination. During a growth cycle, once the spore germination is inhibited or stopped, the spore can no longer form mature mycelium, and finally interrupts pathogen reproduction and terminates the infection cycle [31].

## 4. Materials and Methods

### 4.1. Chemicals

MWCNTs with –OH, –COOH, and –NH_2_ surface functional groups were purchased from ChengDu Organic Chemicals Co., Ltd. Chinese Academy of Science (Chengdu, China) and used as received. The physical dimensions, purity, and functional groups are summarized in Table 1. All CNTs suspensions were obtained by sonication for 30 min using a bath sonicator (Elamsonic, S60H, ELMA-Tech, Mosbach, Germany) at 37 kHz under 550 W without any dispersant.

### 4.2. Fungal Strains

*F. graminearum* was obtained from the State Key Laboratory of Agricultural Microbiology of Huazhong Agricultural University (Wuhan, China). *F. graminearum* spores were obtained as described previously [19]. Briefly, spores incubated in 3% green bean soup liquid medium for 5 days were harvested by centrifugation at 3500 rpm for 5 min. After filtering through gauze, the spores were washed twice with sterile distilled water and adjusted to 5 × 10^5^ spores mL^−1^.

### 4.3. Spore Germination and CNTs Treatment

For spore germination studies, *F. graminearum* spores were treated with CNTs according to a previous report [19]. Briefly, an 80 µL suspension of spores was mixed with 80 μL of different types of MWCNTs (–OH, –COOH, and –NH_2_) in the tubes to obtain MWCNTs at a final concentration of 62.5, 125, 250, and 500 μg·mL^−1^, respectively. Control samples containing 80 µL suspensions of spores were mixed with 80 µL DI water. The 30 µL mixture with a different concentration of MWCNTs was transferred onto a concave slide for further incubation at 28 °C for 5 h in complete darkness. Five concave slides were prepared for each treatment, and the mean germination values were calculated based on five measurements. Photomicrographs were taken with a digital camera connected to a Leica microscope. For each treatment, one hundred spores were used to measure the average spore length. The spore germination rate was calculated as follows:

*R_germ_* = *N_germ_*/*N_total_* × 100%.

*R_germ_* represents the spore germination rate (%); *N_germ_* represents the number of germinated spores; *N_total_* is the total number of spores.

### 4.4. Morphological Observation by TEM

The morphological changes of spores were further investigated using a transmission electron microscopy (TEM) (FEI, Brno, Czech Republic). The spore preparation for morphological study was according to our previous report [19].

### 4.5. Statistical Analysis

Each treatment was performed in four replicates, and the data for all figures were represented as mean values ± SE (standard errors). Statistical analysis used SAS 8.1 software, and the statistical significance was determined by a *p* value < 0.05 (or < 0.01) in a Student’s *t*-test.

## 5. Conclusions

In summary, we studied the effects of MWCNT surface modification on their antifungal activity. It was demonstrated that MWCNTs functionalized with –COOH, –OH, and –NH_2_ groups had a more effective and durable inhibitory antifungal activity than pristine nanotubes. In addition to having an improved solubility, it is likely that the functional groups conjugated to the nanotubes favors interaction with spores, which endows enhanced antifungal activity to the surface modified MWCNTs. Besides this, the effect of length was also studied and was found that it is not a noticeable factor in antifungal activity. Taken together with our preliminary results, this work clearly shows the great potential of these easily produced carbon materials in eliminating a severe agricultural problem.

## Figures and Tables

**Table 1:** Purity, functional groups, and physical dimensions of MWCNTs.

Items	Purity (%)	Functional Groups Content (%)	ID (nm)	OD (nm)	Length (μm)
MWCNTs-OH	>95	3.70	3–5	8–15	~50
MWCNTs-NH_2_	>95	0.45	3–5	8–15	~50
MWCNTs-COOH_1_	>95	3.82	3–5	8–15	~50
MWCNTs-COOH_2_	>95	2.56	2–5	<8	10–30
MWCNTs-COOH_3_	>95	3.86	2–5	<8	0.2–2

This table is provided by ChengDu Organic Chemicals Co., Ltd.

## Supplementary Materials

The following are available online at www.mdpi.com/1996-1944/10/12/1375/s1, Figure S1: Microscopic images of MWCNTs on germination rate of spores, Figure S2: Microscopic images of MWCNTs on germination pattern of spores.
