# Supplementary material for: Surface Modification of Carbon Nanotubes with an Enhanced Antifungal Activity for the Control of Plant Fungal Pathogen
Source: Materials (Basel). 2017 Nov 30;10(12):1375. doi: 10.3390/ma10121375 (PMC5744310; doi:10.3390/ma10121375)
Supplement: Supplementary file 1 [file materials-10-01375-s001.pdf]

materials

Supplementary

Surface Modification of Carbon Nanotubes with an Enhanced Antifungal Activity for the Control of Plant Fungal Pathogen

Xiuping Wang [1], Zilin Zhou [2] and Fangfang Chen [2,*]

[1] College of Life Science and Technology, Hebei Normal University of Science and Technology, Qinhuangdao 066000, China; wangxiuping0721@163.com

[2] CAS Key Laboratory of Plant Germplasm Enhancement and Specialty Agriculture, Wuhan Botanical Garden, Chinese Academy of Sciences, Wuhan 430074, China; zhouzilin16@mails.ucas.ac.cn

* Correspondence: chenff@wbgcas.cn

Received: 3 November 2017; Accepted: 28 November 2017; Published: 30 November 2017

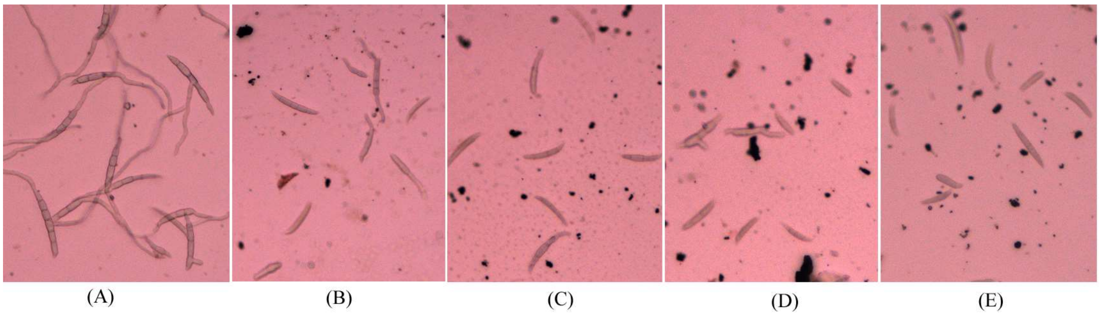

Figure S1. Microscopic images of MWCNTs on germination rate of spores. Spores were germinated on distilled water at 28 °C in darkness (**A**) and at 500 μg mL^{-1} of (**B**) MWCNTs, (**C**) MWCNTs-COOH, (**D**) MWCNTs-OH and (**E**) MWCNTs-NH_2 dispersions. Germination was evaluated after incubation for 5 h. Error bars represent the standard deviation (N = 4).

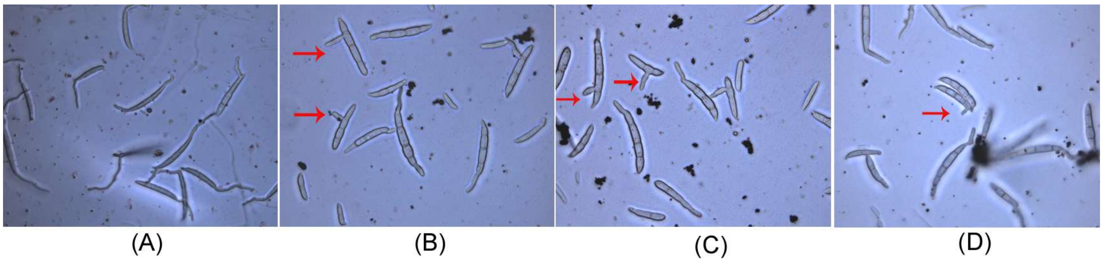

Figure S2. Microscopic images of MWCNTs on germination pattern of spores. (**A**) spores untreated and (**B–D**) treated with 500 μg·mL^{-1} of MWCNTs-COOH, MWCNTs-OH and MWCNTs-NH_2, respectively. The red arrows indicate the germ tubes of the spores developed from the side of spores.
